# A signature-based machine learning model for distinguishing bipolar disorder and borderline personality disorder

**DOI:** 10.1038/s41398-018-0334-0

**Published:** 2018-12-13

**Authors:** Imanol Perez Arribas, Guy M. Goodwin, John R. Geddes, Terry Lyons, Kate E. A. Saunders

**Affiliations:** 1 Mathematical Institute, University of Oxford, Oxford, UK; 2 Department of Psychiatry, University of Oxford, Oxford, UK; 3 Oxford Health NHS Foundation Trust, Oxford, UK; 4 NIHR Oxford Health Biomedical Research Centre, Oxford, UK; 5 Alan Turing Institute, London, UK

## Abstract

Mobile technologies offer new opportunities for prospective, high resolution monitoring of long-term health conditions. The opportunities seem of particular promise in psychiatry where diagnoses often rely on retrospective and subjective recall of mood states. However, deriving clinically meaningful information from the complex time series data these technologies present is challenging, and the current implications for patient care are uncertain. In this study, 130 participants with bipolar disorder (*n* = 48) or borderline personality disorder (*n* = 31) and healthy volunteers (*n* = 51) completed daily mood ratings using a bespoke smartphone app for up to 1 year. A signature-based learning method was used to capture the evolving interrelationships between the different elements of mood and exploit this information to classify participants’ diagnosis and to predict subsequent mood. The three participant groups could be distinguished from one another on the basis of self-reported mood using the signature methodology. The methodology classified 75% of participants into the correct diagnostic group compared with 54% using standard approaches. Subsequent mood ratings were correctly predicted with >70% accuracy. Prediction of mood was most accurate in healthy volunteers (89–98%) compared to bipolar disorder (82–90%) and borderline personality disorder (70–78%). The signature method provided an effective approach to the analysis of mood data both in terms of diagnostic classification and prediction of future mood. It also highlighted the differing predictability and the overlap inherent within disorders. The three cohorts offered internally consistent but distinct patterns of mood interaction in their reporting which have the potential to enable more efficient and accurate diagnoses and thus earlier treatment.

## Introduction

Historically the diagnosis of psychiatric disorders has been hampered by the inherent inaccuracy of retrospective narrative recall of abnormal mental states and the difficulty of defining their persistence over time. Furthermore, diagnostic classifications have changed little since the original description made by Kraepelin and are not clearly related to the underlying biology, which remains unknown^1^. The shortcomings of current diagnostic categories have motivated attempts (notably the NIMH Research Domain Criteria [‘RDoC’]) to take a radical and ‘bottom up’ approach to diagnosis. In recent years, the rapid development of mobile technologies has allowed more precise measures of subjective psychopathology. However, analysis of these data streams has been challenging. Often the data generated by these measurements is sequential in nature, where the most valuable information is contained in the order at which different events occur, rather than in any individual event. These types of data need an analytic approach that exploits this distinctive feature. Signatures from rough path theory^2^ have proven to be an effective way of analysing these streams of data since they naturally capture the order of events. As a consequence, signatures have successfully been applied to a number of machine learning problems where the object that is being studied is a stream of data. For instance, using the path signature as the core feature representation for handwritten sequential data, Dr. B. Graham won the ICDAR 2013 international challenge on online Chinese character recognition^3^. The effectiveness of signatures in data science has led to a number of publications where a signature-based approach has achieved better performance relative to approaches that do not use signatures, in areas such as handwriting recognition, gesture recognition and action detection^4–12^.

Bipolar disorder (BD) and borderline personality disorder (BPD) are common mental disorders^13–15^. BD is a mood disorder with a strong genetic basis while BPD is a disorder of personality commonly related to abusive experiences in childhood. Although the two disorders are thought to develop through different processes and mechanisms, BD and BPD can be difficult to differentiate clinically as both are characterised by mood instability and impulsivity, and the behavioural consequences can be similar^16^. However, correct diagnosis is essential because of the contrasting treatment approaches to the two disorders^17,18^.

Here, we use a signature-based machine learning model to re-analyse data obtained from a clinical study^19^, which explored daily reporting of mood in participants with bipolar disorder, borderline personality disorder and healthy volunteers. Specifically, we sought to classify the diagnosis of participants on the basis of their evolving mood and also predict their mood the following day. The generality of the signature-based machine learning model allows these problems to be treated in similar ways.

**Table 1:** Accuracy and area under the ROC curve

	Healthy		Bipolar		Borderline	
	Accuracy	AUC	Accuracy	AUC	Accuracy	AUC
Healthy			84%	0.91	93%	0.98
Bipolar					80%	0.86
Borderline						

## Materials and methods

### Data

Prospective mood data was captured from 139 individuals who were taking part in the AMoSS study. Of the 139 recruited participants, nine participants who withdrew consent or failed to provide at least 2 months of data were excluded from further analysis, so the data set consists of 130 individuals in total. The AMoSS study was a prospective longitudinal study during which used a range of wearables in combination with a bespoke smartphone app to better characterise mood instability and its correlates in bipolar disorder (*n* = 48), borderline personality disorder (*n* = 31) and healthy volunteers (*n* = 51). Participants rated their mood daily across six different categories (anxiety, elation, sadness, anger, irritability and energy) using a 7-point Likert scale (with values from 1 (not at all) to 7 (very much)). These mood and symptom states were based on previous work done in the context of the OXTEXT study. Data were collected from each participant for a minimum of 3 months, although 61 of the 130 participants provided data for more than 12 months. Overall compliance was 81.2%. The number of male participants in the study was 16 for BP, 2 for BPD and 18 for healthy controls, of ages 38 ± 21 (BP), 34 ± 15 (BPD) and 37 ± 20 (healthy volunteers). Forty-seven of the BP participants and 23 of the BPD participants were taking psychotropic medication. Healthy participants were all medication free (Table 2). An analysis of this data set has previously been published by Tsanas et al.^20^.

**Table 2:** Demographic characteristics of the three groups. Where appropriate distributions are summarised in the form of the median +/- the interquartile range

	Bipolar disorders (BD)	Borderline personality disorders (BPD)	Healthy controls (HC)
Originally recruited	53	33	53
Processed data from	48	31	51
Days in study	353±261	313±107	276±253
Age (years)	38±21	34±15	37±20
Gender (males)	16	2	18
Any psychotropic medication	47	23	0
Lithium	19	0	0
Anticonvulsant	19	1	0
Antipsychotic	33	6	0
Antidepressants	17	23	0
Hypnotics	3	2	0

The mood data was divided into *buckets* comprised of streams of 20 consecutive self-reported mood states. Although these observations were typically recorded daily, data did not have to come from 20 consecutive days. Thus, streams could be constructed even when participants failed to record their mood on some days or when participants recorded their mood several times per day. This generated 2192 buckets of streamed data. These were randomly separated into a training set of 1534, and a testing set of 658 streams of data. We then normalised the data to capture the intrinsic properties of the data independent of arbitrary units such as the scale used to record the scores or the period of time when the study was carried out.

The stream of mood reports was identified as a seven-dimensional path (one dimension for time, six dimensions for the scores) defined on the unit interval.

**Fig. 1:**
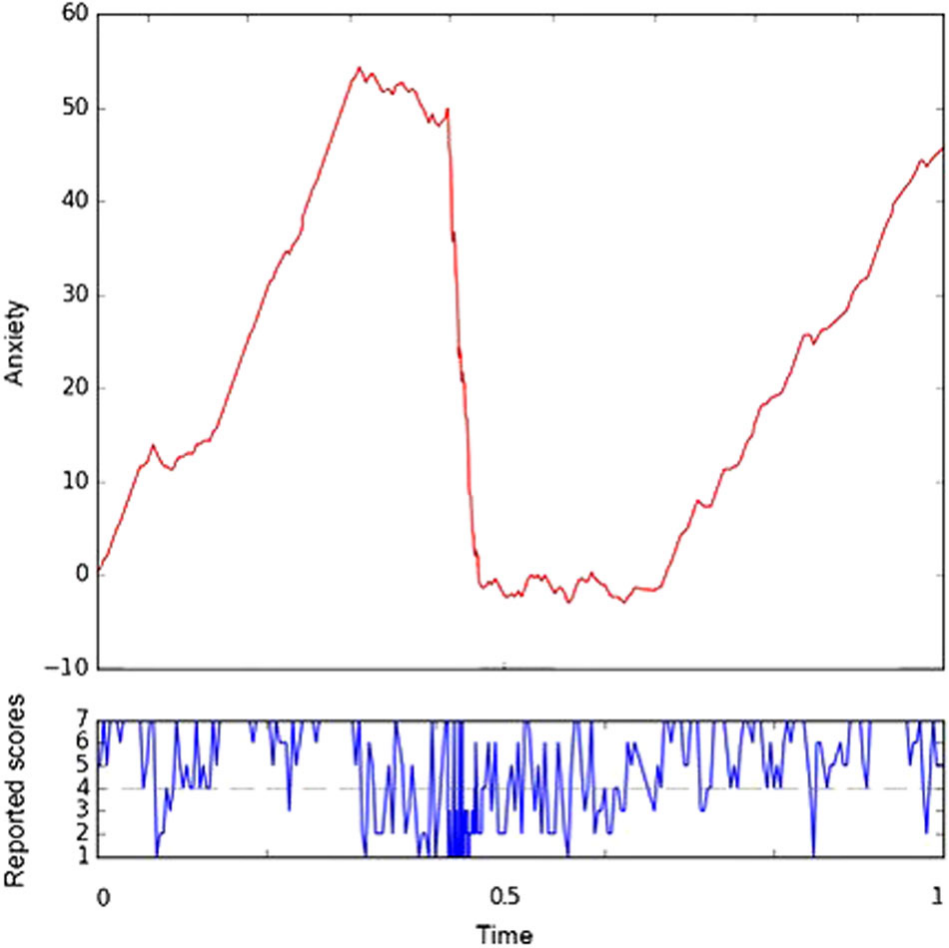
Normalised anxiety scores of a participant with bipolar disorder (above), which were calculated by aggregating the reported scores (below and centred) plotted against normalised time. As we see, high levels of reported scores correspond to upward trends, low levels of reported scores correspond to downward trends and periods of time of high oscillations in the reported scores are represented by oscillations in the path

Figure 1 shows the aggregated normalised anxiety scores of a participant with bipolar disorder plotted against normalised time.

**Fig. 2:**
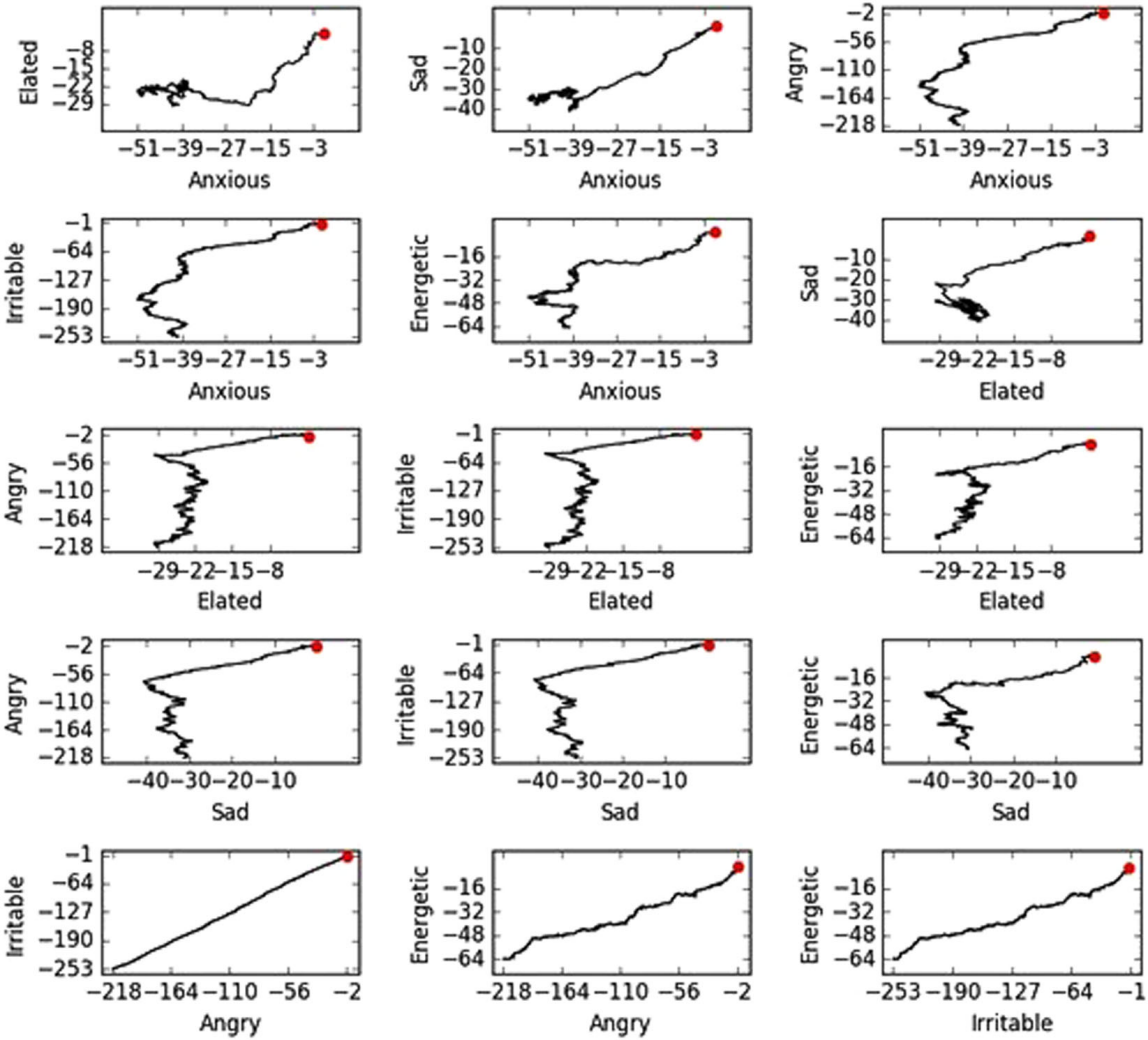
Normalised scores of each category plotted against all other categories, for a participant with bipolar disorder. The red point indicates the starting point. Notice that the scale is different in each plot

Figure 2 shows the normalised scores of each category plotted against all other categories. This way of representing the path allows interpretation of the order at which the participant changes mood. Take for example, the Angry vs. Elated plot (third row, first column). The path starts at the point (0, 0). As we see, the path moves left first. Therefore, the participant is becoming less and less elated, while the score in anger remains approximately constant. Suddenly, the period of low elation stops, and the participant starts recording low scores in anger. This low level in anger remains persistent for the rest of the path. On the other hand, if one considers the Angry vs. Irritable plot (fifth row, first column), we observe that it is essentially a straight line and the levels of anger and irritability match closely for this participant.

### Group classification

We started by establishing a set of input-output pairs {(*R*_*i*_,*Y*_*i*_)}_*i*_, where *R*_*i*_ is the seven-dimensional path of aggregated normalised scores for participant *i*, as described above, and *Y*_*i*_ denotes the group it was diagnosed into at the beginning of the study. This set was transformed into a new set of input/output pairs, {(*S*^*n*^(*R*_*i*_),*Y*_*i*_)}_*i*_ where *S*^*n*^(*R*_*i*_) denotes the truncated signature^2^ of order *n* of the stream *R*_*i*_. The truncated signature is a feature set describing the stream whose size depends on the truncation degree but not on the number of sample points (or indeed the paramaterisation) of the stream. The signature of order *n* grows exponentially with *n*, and therefore large values of *n* will produce input vectors of large dimensions but far smaller than other feature sets where the sampling rate directly changes the dimension of the feature set. We have only considered feature sets based on truncated signatures up to degree *n* = *1, 2, 3, 4*. Random forest^21^ was used to learn the mapping between the input and the output.

Given that it has already been established that there are differences in mean mood scores between the groups overall^20^ we also calculated the mean score in each mood category and classified streams of 20 consecutive observations on this basis as a comparison to the signature method. To assess the performance of the classification procedure we computed accuracy, sensitivity, specificity, positive predicted value (PPV). We also trained the model using pairs of clinical groups and computing the area under the receiver operating characteristic curve (ROC) to assess the performance of the classification model at different values of threshold. Since this was done for each pair of clinical groups, we obtained a matrix with different values of the AUC. Moreover, in order to have a better understanding of how robust these percentages are, we applied bootstrapping to the model with the signature order fixed to 2.

We also considered the extent to which participants were characterised as belonging to each specific clinical group. We trained the model using all participants except the one we were interested in, and then tested the model with 20-observations periods from this person. We calculated the proportion of periods of time when the participant was classified as bipolar, healthy or borderline, which allowed us to plot the participant as a point in the triangle. This process was followed for every participant, although we excluded participants that generated <5 buckets of 20 observations in order to avoid extreme values for them. 121 participants were included.

### Predicting the mood of a participant

In this case, the aim was to predict the mood of a participant, using the last 20 observations of the participant. We constructed the input-output pairs {(*R*_*i*_,*Y*_*i*_)}_*i*_ where *R*_*i*_ was the normalised seven-dimensional path of a particular participant, and *Y*_*i*_∈{1,...,7}^6^ was the mood of the participant the next observation he or she registered. We then constructed a new set of input-output pairs {(*S*^*n*^(*R*_*i*_),*Y*_*i*_)}_*i*_ with *S*^*n*^(*R*_*i*_) the truncated signature of order *n*. We applied regression using random forest to these pairs of inputs and outputs, thus obtaining our model. The model was trained using data from each clinical group separately.

To measure the accuracy of the predictions, we used the mean absolute error (MAE) and the percentage of correct predictions. A prediction $\hat y \in \left\{ {1,...,7} \right\}$ was considered correct if $|\hat y - y| \le 1$, where *y* is the correct score.

We used the publicly available Python eSig package (version 0.6.31) to calculate signatures of streams of data, Python pandas package (version 0.20.1) for statistical analysis, data manipulations and processing, Python scikit-learn package (version 0.18.1) for machine learning tasks and matplotlib for plotting and graphics (version 2.0.1).

The study was approved by the NRES Committee East of England—Norfolk (13/EE/0288) and the Research and Development department of Oxford Health NHS Foundation Trust.

## Results

### Predicting group membership

The signature-based model categorised 75% of participants into the correct diagnostic group when the second-order signature was considered (*n* = 2, where *n* denotes the order of the signature). When the order was increased, the accuracy dropped slightly (70% for *n* = 3 and 69% for *n* = 4). The signature method performed significantly better than the naive model using the mean score in each category over the 20 observations, which classified just 54% of participants correctly. On bootstrapping the accuracy of the second-order signature was 74.85% while the standard deviation was 2.05, suggesting that the results are stable and robust. Table 1 shows the accuracy and area under the ROC curve when only pairs of diagnosis are considered for classification, rather than all three groups.

The signature of order 2 was able to capture second-order effects on the stream of data and since the feature set has 57 dimensions it was reasonable to learn linear relations from the 517 buckets of data in the training set. To capture the full second-order effects using raw streams of data, the feature set would have required over 10,000 dimensions. A 57-dimensional feature set of randomly selected quadratic polynomials on the raw data stream was inferior to the signature model: the average accuracy was 68% for the random feature set, well below the 75% accuracy of the second-order signature model.

**Fig. 3:**
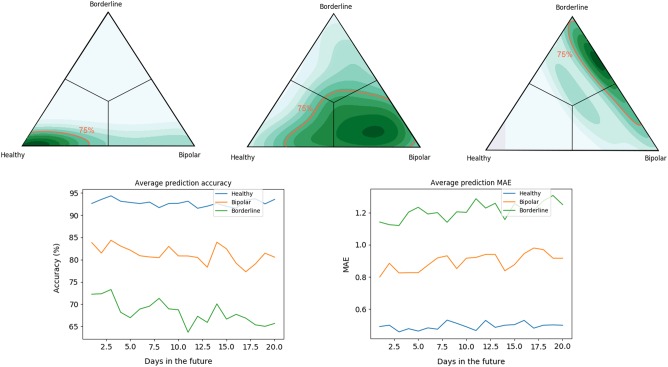
Diagnosis classification and multiperiod mood prediction. **a** Healthy participants, **b** Bipolar participants and **c** Borderline participants. Bottom: Decay in accuracy (left) and MAE (right) of the mood predictions for the three clinical groups, when the prediction horizon is increased

The proportion of a participant’s sequential mood buckets matched each specific diagnostic group and positioned that specific individual on a triangular spectrum; the distribution of that density for each initial diagnosis is shown in Fig. 3 (top). In each of the plots, the regions of highest density of participants are located in the correct corner of the triangle: the greatest consistency is with the healthy participants.

Table 3 shows that the signature-based model clearly distinguishes healthy participants from the clinical groups, obtaining an accuracy of 84% in classifying BD participants and HC, and 93% in classifying BPD participants and HC.

**Table 3:** Summary of the future mood prediction accuracy

	Bipolar		Borderline		Healthy	
	Accuracy	MAE	Accuracy	MAE	Accuracy	MAE
Anxious	82%	0.96	73%	1.17	98%	0.4
Elated	86%	0.75	78%	1.03	89%	0.57
Sad	84%	0.77	70%	1.16	93%	0.41
Angry	90%	0.60	70%	1.12	98%	0.30
Irritable	84%	0.84	70%	1.15	97%	0.39
Energetic	82%	0.90	75%	1.00	89%	0.69

### Predicting the mood of an individual participant

Using 20 consecutive observations, the signature-based model correctly predicted the subsequent mood score in healthy participants with 89–98% accuracy. In bipolar participants the mood was correctly predicted 82–90% of the time while in borderline participants this was 70–78% of the time (see Table 3). Moreover, the differences in the accuracy of predictions across the three clinical groups highlight a clear separation between the diagnostic groups, suggesting that the three clinical groups have intrinsically different predictive relationship between reported and future mood state.

We compared our model to the naive benchmark that predicts that next day’s score will be equal to the last day’s score. In this case, the mood of healthy participants was correctly predicted 61–92% of the time, while the mood of bipolar and borderline participants was correctly predicted 46–67% and 44–62% of the time. Our model clearly outperformed this benchmark.

We then explored the possibility of predicting the change of mood over different time horizons. The results are shown in Fig. 3 (bottom). The predictability of the mood changes decay when the time horizon is increased. The most significant decay in predictability was found in BPD participants, consistent with the unpredictability that characterises BPD.

## Discussion

This analysis uses the signature feature set combined with random forests to study self-reported mood data. The signature-based model, with its ability to ignore parameterisation and provide canonical low dimensional sets of features, proved to be effective in distinguishing the three participant groups on the basis of their self-reported mood and was more accurate in doing so than previously used metrics, specifically mean mood scores. Furthermore, the method showed a clear overlap between the HC and BD groups suggestive of a normalisation of mood structure in some BD participants for some of the time; this confirms clinical experience. An alternative explanation could be that the six self-reported mood categories were not sensitive to the abnormal mood states associated with bipolar disorder.

By contrast there is a very clear differentiation between HC and BPD. This finding supports the concept of BPD as a distinct disorder. The distinctness from the healthy controls makes it more difficult to conceptualise BPD as an extreme variation of normal personality. There was some overlap between BD and BPD, but also marked differences. Overall the signatures of mood in the BPD group do appear to be distinct from both health and bipolar groups. The findings have implications for diagnostic practice where rates of misdiagnosis of BPD and BD are significant^22,23^. There is previous work published on machine learning approaches to distinguishing BD and BPD from healthy controls. For instance, some publications^22,23^ report that MRI data can be used to classify individuals with borderline personality disorder and healthy controls, obtaining an accuracy of and 80%^24^ and 93.55%^25^, although the latter percentage should be taken with caution due to the small sample size. Neuroimaging has also been used to detect bipolar^26^ disorder with an accuracy of 59%, as well as other sources of data such as verbal fluency^27^ with an accuracy of 79%. Therefore our approach has improved existing results, although comparison across the different papers is difficult due to the different datasets that were used.

The ability of the signature methods to predict future mood on the basis of the 20 preceding observations is significantly better than the used benchmark and again highlights the way in which the three groups can clearly be distinguished on the basis of the predictability of their mood states. The ability to predict future mood states is a key component of proactive self-management in BD and may be the key to preventing major depressive or manic relapse. While the predictability of mood in BPD was less accurate it was still in the order of 70%-78%. The unpredictable nature of mood in BPD is a characteristic feature of the disorder but the signature approach indicates that this lived experience may be more computationally predictable than has previously been thought. Further analyses and larger data sets, as well as exploration of different frequencies of measurement are required to explore whether the predictive accuracy of future mood states is maintained for more distant time points.

Our results suggest that the second-order signature is the most effective one given the available data. The signature of second-order is able to capture second-order effects across pairs of moods in a meaningful and concise way. If the same effect wanted to be obtained using the raw parameterised seven-dimensional path, we would have an initial feature set of more than 10,000 dimensions—and to train usefully would have required a much larger data set than our 733 instances. It may also be informative to enhance the mood data with automatically generated data derived from mobile phones, for example, voice^28^ or geolocation^29^.

While it requires replication in other cohorts, and specifically in cohorts recruited independent of diagnosis, the signature method offers an exciting approach to the analysis of mood data. Despite the promise of time-stamped longitudinal symptom data the analytic challenge has been considerable and to date there is little consensus as to which approach to use. The signature method adds to the toolkit of analytic approaches and is particularly well suited to complex times series mood ratings because of its ability to capture order and deal with missing data.

### Limitations

The study population was derived from patient samples but did not include those who were acutely unwell or who had significant comorbidities and as such may have represented a more stable sub-population of patients. We had relatively little contact with patients during the study and only a baseline clinical assessment was carried out such that the validity of the self-reported mood scores cannot be certain. However, we have no evidence to suggest that the reports are inaccurate and they are likely to be a good representation of the patients lived experience. This is supported by the fact that, as shown in this paper, the self-reported scores were significant enough to meaningfully distinguish the three groups. We also kept no ongoing record of medication changes through the duration of the study.

Our data set consisted of 733 examples. The data set was quite small for machine learning purposes and so we avoided testing different models in order to avoid overfitting. A bigger data set would undoubtedly allow more flexibility.

## Disclaimer

The views expressed in this publication are those of the authors and not necessarily those of the NIHR, the Medical Research Council, the National Health Service or the Department of Health.
